# Consecutive magnetic resonance imaging during brachytherapy for cervical carcinoma: predictive value of volume measurements with respect to persistent disease and prognosis

**DOI:** 10.1186/s13014-015-0559-5

**Published:** 2015-12-08

**Authors:** J. E. Mongula, B. F. M. Slangen, D. M. J. Lambregts, F. Cellini, F. C. H. Bakers, L. C. H. W. Lutgens, T. Van Gorp, A.J. Kruse, R. F. P. M. Kruitwagen, R. G. H. Beets-Tan

**Affiliations:** Department of Obstetrics & Gynaecology, Maastricht University Medical Centre, Postbus 5800, 6202 AZ Maastricht, The Netherlands; GROW, School for Oncology and Developmental Biology, Maastricht, The Netherlands; Department of Radiology, Maastricht University Medical Centre, Maastricht, The Netherlands; MAASTRO clinic, Postbus 3035, 6202 NA Maastricht, The Netherlands; Department of Radiology, The Netherlands Cancer Institute, Amsterdam, The Netherlands

**Keywords:** Cervical carcinoma, Brachytherapy, Magnetic resonance imaging, Tumor volume, Predictive tool

## Abstract

**Background:**

Cervical cancer is associated with a high yearly mortality. The presence of persistent disease after radiotherapy is a significant predictor of patient survival.

The aim of our study was to assess if tumor volume regression measured with MR imaging at the time of brachytherapy can discriminate between patients who eventually will achieve a complete response to radiotherapy from those who will not. The second objective was to evaluate whether tumor volume regression predicts overall treatment failure.

**Methods:**

MRI was evaluated quantitatively in 35 patients; by means of tumor volumetry on T2-weighted MR images before treatment, at the first BCT application, and at the final BCT. The MR images were independently analyzed by two investigators. As a reference standard histopathologic confirmation of residual tumor and/or clinical exam during follow-up > 1 year were used. Area under the curve were compared, *P*-values <0.05 were considered significant.

**Results:**

There was a good correlation between volume measurements made by the two observers. A residual tumor volume >9.4 cm^3^ at final BCT and tumor volume regression < 77 % of the pre-treatment volume were significantly associated with local residual tumor after completion of therapy (*p* < 0.02) (AUC, 0.98-1.00). A volume >2.8 cm^3^ at final BCT was associated with overall treatment failure (*p* < 0.03).

**Conclusion:**

Our study shows that volume analysis during BCT is a predictive tool for local tumor response and overall treatment outcome. The potential of local response assessment to identify patients at high risk of overall treatment failure is promising.

## Background

Cervical cancer is associated with a yearly mortality of 270.000 patients [1–3]. The presence of a persistent cervical carcinoma after radiotherapy is a significant predictor of patient survival. In a select group of patients with persistent tumors after radiotherapy, surgical resection can be beneficial [4, 5].

The detection of residual tumors by gynecological examination, MRI, and PET-CT is complicated by the occurrence of false positive results and interobserver dependency, which are mainly due to post-radiation induced fibrosis. Diagnoses based on histological biopsies are limited by the occurrence of false negative results [6–9]. It has been reported that tumor volume assessment on repeated MRIs during radiotherapy treatment can be used to predict tumor response to radiotherapy [10–13]. In the past decade a new concept was clinically introduced by the GEC-ESTRO working group taking the tumor regression during external beam radiation into account for treatment planning with brachytherapy (BCT) [14]. Applying this concept, i.e., MR-image guided adaptive brachytherapy (IGABT), assessing the residual tumor volume and/or target volume at high risk at the time of BCT has been demonstrated to improve outcome significantly in locally advanced cervix carcinoma [14–16]. For optimal assessment of residual tumor burden at the time of BCT T2-weighted MR images are obtained with the BCT applicator in place. This imaging sequence results in the best quality to discriminate tumor from cervical stroma. Applicator based distortions and artefacts are acceptable for T2W sequences using MR-compatible applicators [17].

Until now, volumetric analysis for predicting treatment response on MRI used in this setting, with the BCT applicator in place, has never been used. If MR-based tumor volume assessment at the time of BCT is a reliable option, it could be used to predict tumor response both local and for overall outcome.

The aim of our study was to assess if absolute tumor volume and tumor regression at the time of BCT can discriminate between patients who eventually achieve a complete response from those who do not. The second objective was to evaluate whether local volume regression can predict overall treatment failure.

## Methods and materials

### Patients

We performed a retrospective analysis of patients with primary cervical cancer (International Federation of Gynecology and Obstetrics (FIGO) stages ≥ IB), who were referred to our center (Maastricht University Medical Centre en MAASTRO clinic) between March 2008 and August 2010 for radiotherapy. Inclusion criteria consisted of: (1) a histologically proven primary cervical carcinoma; (2) availability of MRI images before treatment (a), after EBRT at the first BCT application (b) and at the last BCT treatment (c); (3) treatment consisting of EBRT (46.0–50.4 Gy and high-dose-rate BCT, with or without concomitant chemotherapy (CT) or hyperthermia (HT); and (4) absence of distant metastasis at diagnosis.

### Radiotherapy protocol

For external beam radiation (EBRT) a dose of 45-50.4 Gy was delivered in 25-28 daily fractions, 5 days a week. Dose specification and homogeneity requirements were according to ICRU-50. In case of concomitant chemotherapy or regional hyperthermia, these modalities were applied once-weekly during EBRT. The first BCT was scheduled in the final week of EBRT of following EBRT. For brachytherapy MR-compatible applicators (Nucletron) were used placing an intra-uterine tandem and vaginal ovoids with or without the use of interstitial needles. For BCT planning the GEC-ESTRO guidelines were followed, contouring the gross tumor, a high-risk (HR) and intermediate risk clinical target volume (IR-CTV) and the organs at risk. Typically 3 applicator insertions were performed under general anesthesia with one week interval. For the second BCT procedure the applicator was left in place for one night allowing 2 BCT treatments with an interval of at least 15 h. Hence, 4 BCT treatments were delivered in 2 weeks. A dose of 7 Gy per fraction was aimed to prescribe to the HR-CTV, aiming to deliver a D90 ≥ 85 Gy.

### MRI protocol

MRI was performed with a 1.5-Tesla MRI unit (Intera (Achieva); Philips Healthcare, Best, The Netherlands or Siemens Magnetom Avanto, Erlangen, Germany) in 52 % of the studies and with a 3.0-Tesla MRI unit (Achieva 3.0TX; Philips Healthcare, Best, the Netherlands) in 48 % of the studies. Patients were placed in a feet-first supine position. The protocol consisted of 2D T2-weighted (T2W) fast spin-echo images (TR/TE 3200–5830/122–150 msec, 18–24 ETL, 3–4 NSA, 0.98 × 0.98 × (3.5–5.0) mm^3^ voxel at 1.5 T and TR/TE 7000/150, 28 ETL, 2 NSA, 0.98 × 0.98 × (3.00–4.00) mm^3^ voxel at 3 T). Scans were made in three planes (sagittal, axial, and coronal), while the axial and coronal planes were angled perpendicular and parallel to the cervical axis, respectively. Patients did not receive bowel preparation or anti-spasmodic agents. MRI was performed at different time points: before treatment (a), after EBRT at the first BCT application (b), and at the final BCT treatment (c). All MRI scans (except the first one, before the onset of radiotherapy) were performed immediately before the BCT fractions were given but with the BCT applicator in situ.

### Image evaluation

MRI was evaluated quantitatively; by means of tumor volumetry on T2-weighted MR images before treatment (a), after EBRT at the first BCT application (b), and at the final BCT treatment (c).

**Fig. 1:**
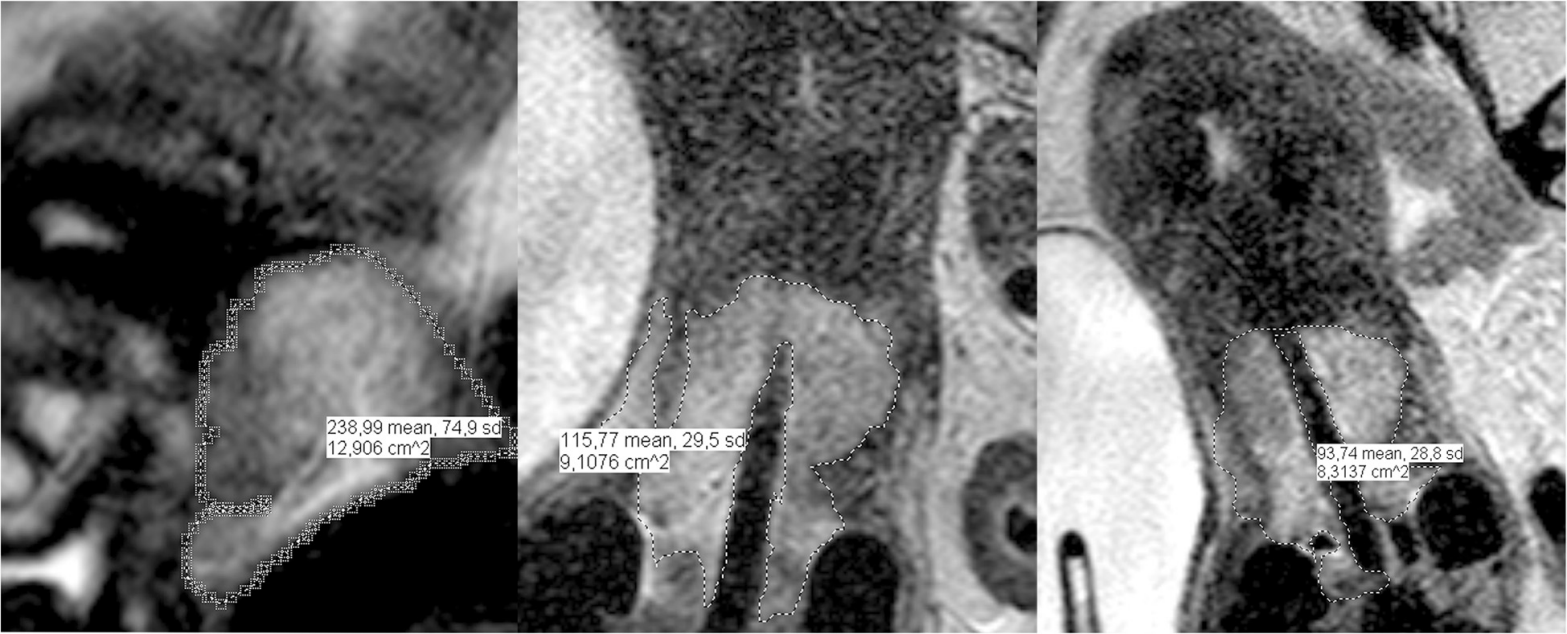
Volume measurement of cervical carcinoma, pre-treatment, at first and final BCT

The MR images were independently analyzed by the investigator (JEM) and a radiation oncologist (FC) blinded to patient information and patient outcome. Volumetric measurements of the tumors on the MR images at all three time points were performed by the two readers, independently, and the readers were blinded to each other’s results. All measurements were performed on a DICOM based system for diagnostic images, IntelliSpace PACS, (Philips Healthcare, Best, The Netherlands). Using these measurements, a suspected tumor was defined as an isointense mass within the irradiated tumor bed in concordance with the GEC-ESTRO guidelines for depicting gross tumor volume. Tumor volume was calculated by placing freehand regions of interest along the border of the suspected tumor on T2W sagittal or transversal MR images, to obtain the sectional tumor area of each tumor-containing slice, and multiplying the sectional tumor area obtained with section thickness [18] (Fig. 1).

The tumor volume reduction (Δvolume) was calculated as the absolute volume obtained from the post-treatment MRI scan minus the initial tumor volume obtained from the pre-treatment MRI scan divided by the initial tumor volume obtained from the pre-treatment MRI scan: [18] ((post-volume – pre-volume)/pre-volume) *100.

### Standard of reference

The presence or absence of a local residual tumor (in cervix and/or vagina, parametria, bladder, or rectum) was determined by:

1. histopathology of the surgical resection specimen (*n* = 11);
2. the results of a post-treatment gynecologic examination (under anesthesia, with or without biopsy) performed 3 months after completion of the entire radiation treatment and after at least 12 months of documented follow-up (*n* = 24).

The presence of distant metastases was proven either by histopathology or the detection of a growing tumor mass during subsequent imaging analysis. Overall treatment failure was defined as the presence of local residual or recurrent tumor (histologically proven), distant metastases, or both.

### Statistical analysis

Statistical analyses were performed using SPSS Statistics v18.0 (SPSS Inc, Chicago, Ill.) and Stata v11.0 (StataCorp LP, Texas). Interobserver variations were assessed by means of interclass correlation coefficients for single and average measures.

Receiver operating characteristics (ROC) curves were constructed to evaluate diagnostic performance for *(a)* absolute MRI volume measurements and *(b)* volume regression (Δvolume). Corresponding areas under the ROC curve (AUC), sensitivity, specificity, and positive and negative predictive values were calculated according to the optimal cut off, determined according to the point nearest to the upper left corner in the ROC curve. Sensitivities and specificities for the different scoring methods were compared using the McNemar test for paired data or the chi-squared test for unpaired data. AUCs were compared according to the method described by De Long et al. [19].

*P*-values less than 0.05 were considered statistically significant.

## Results

A total of 48 consecutive patients were identified, of which 35 met the inclusion criteria. Their baseline characteristics are presented in Table 1.

**Table 1:** Baseline Characteristics

Age median(range)	53 years (32–77)
Histology	
Squamous cell carcinoma	28 (80 %)
Adenocarcinoma	5 (14 %)
Other	2 (6 %)
FIGO	
I	
Ib1	5 (14 %)
Ib2	4 (11 %)
II	
IIa	5 (14 %)
IIb	17 (49 %)
III	
IIIa	0
IIIb	2 (6 %)
IV	
IVa	2 (6 %)
Therapy	
Chemo-radiotherapy	29 (83 %)
Hyperthermia-radiotherapy	6 (17 %)

FIGO: The International Federation of Gynecology and Obstetrics

### Patient and treatment characteristics

After treatment, a local residual tumor was histologically proven by biopsy in four patients, of whom one patient had concurrent distant metastases and one patient had concurrent lymphatic metastases. Six patients had distant metastases only. Two had distant or lymphatic metastases. Six patients had distant metastases only. At a median follow-up of 22 months (range, 13–41), the remaining 25 patients were still free of disease.

### MRI volumetry for detection of local residual tumor

The absolute volume measurements of both observers are shown in Table 2. A significant decrease in tumor volume during treatment was observed in all patients (*p* ≤ 0.01). The AUC, interclass correlation coefficients, sensitivity and specificity for absolute volume, and volume regression are shown in Table 3. There was a good correlation between the absolute volume measurements made by the two observers (interclass correlation coefficients, 0.90–0.98).

**Table 2:** Absolute MRI tumor volume measurements and regression before treatment, at first and at final brachytherapy during radiotherapy treatment of cervical cancer

Evaluation	Observer 1		Observer 2	
	volume (cm^3^)	Δvolume(%)	volume (cm^3^)	Δvolume(%)
Local complete response (n:31)				
(a) MRI before treatment	33 (13–104)	-	33 (11–130)	-
(b) MRI at the first BCT*	8.5 (0.4–28)	73 % (48–97)	8 (1–23)	73 % (37–95)
(c) MRI at the final BCT*	3.7 (0–14)	88 % (75–100)	3.3 (0.5–9)	89 % (80–100)
Local residual tumor (n:4)				
(a) MRI before treatment	32 (15–44)	-	28 (19–37)	-
(b) MRI at the first BCT	18 (14–23)	36 % (8–55)	16 (10–23)	39 % (21–68)
(c) MRI at the final BCT	13 (4–15)	53 % (21–67)	11 (3–13)	55 % (28–74)

*p ≤ 0.01 compared to the residual tumor group

**Table 3:** Diagnostic performance of volume measurements for depicting local residual cervical tumor using MRI before treatment, at first and final BCT

Evaluation	AUC		Interclass coefficient (average)	Cut-off	Sensitivity and specificity			
	Observer 1	Observer 2			Observer 1		Observer 2	
					Sensitivity	Specificity	Sensitivity	Specificity
Absolute volume analysis (n:35)								
(a) MRI before treatment	0.60 (0.30–0.90)	0.53 (0.29–0.78)	0.98 (0.96–0.99)	28,6 cm^3^	75 % (22–99)	45 % (28–64)	75 % (22–99)	55 % (36–72)
(b) MRI at the first BCT*	0.91 (0.81–1.00)	0.89 (0.78–1.00)	0.95 (0.91–0.98)	11,5 cm^3^	100 % (40–100)	71 % (52–84)	100 % (40–100)	84 % (69–96)
(c) MRI at the final BCT*	0.98 (0.94–1.00)	1.00 (1.00–1.00)	0.94 (0.89–0.97)	9,4 cm^3^	100 % (40–100)	94 % (77–100)	100 % (40–100)	100 % (86–100)
Volume regression analysis (n:35)								
ΔVolume: First BCT MRI/MRI before treatment	0.98 (0.93–1.00)	0.89 (0.72–1.00)	0.94 (0.87–0.97)	69 %	100 % (40–100)	58 % (39–75)	100 % (40–100)	65 % (45–80)
ΔVolume: Final BCT MRI/MRI before treatment*	1.00 (1.00–1.00)	1.00 (1.00–1.00)	0.97 (0.95–0.99)	77 %	100 % (40–100)	97 % (81–100)	100 % (40–100)	100 % (86–100)

AUC are given with the corresponding 95 % confidence intervals. *p < 0.05, compared to (a)

A tumor volume at first BCT >11.5 cm^3^ predicted local residual tumor following treatment (*p* < 0.03). A volume >9.4 cm^3^ at the final BCT and <77 % volume regression compared to the pre-treatment volumes obtained with MRI (Δvolume) were associated with a local residual tumor after completion of therapy (*p* < 0.02) (AUC, 0.98–1.00).

### MRI volumetry for prediction of overall treatment failure

The interclass correlation coefficients, AUC, and the *p*-values for absolute volume and volume regression are shown in Table 4. A tumor volume >2.8 cm^3^ at the final BCT was statistically associated with overall treatment failure (*p* < 0.03) (AUC, 0.79–0.81). Also, <89 % tumor volume regression (Δvolume) compared to the pre-treatment volume assessed with MRI scan significantly predicted overall treatment failure (AUC 0.87–0.78).

**Table 4:** Diagnostic performance of volume measurements for assessing overall treatment failure using MRI before treatment, at first BCT and final BCT

Evaluation	AUC; Significance		Interclass coefficient (average)	Cut-off
	Observer 1	Observer 2		
Absolute volume analysis (n:35)				
(a) MRI before treatment	0.56 (0.37–0.75); -	0.57 (0.38–0.76); -	0.98 (0.96–0.99)	28,6 cm^3^
(b) MRI at the first BCT	0.82 (0.69–0.96); *p* < 0.01	0.78 (0.63–0.93); *p* = 0.02	0.95 (0.91–0.98)	7,7 cm^3^
(c) MRI at the final BCT	0.81 (0.67–0.96); *p* < 0.01	0.79 (0.63–0.95); *p* = 0.03	0.94 (0.89–0.97)	2,8 cm^3^
Volume regression analysis (n:35)				
First BCT MRI/MRI before treatment	0.87 (0.75–0.99); *p* < 0.01	0.78 (0.72–1.00); *p* = 0.11	0.94 (0.87–0.97)	72 %
Final BCT MRI/MRI before treatment	0.84 (0.69–0.89); *p* = 0.02	0.79 (0.63–0.95); *p* = 0.10	0.97 (0.95–0.99)	89 %

AUC are given with the corresponding 95 % confidence intervals

## Discussion

Our study shows that absolute volume measurements as well as volume regression determination at the time of the final BCT are accurate predictors for treatment outcome. Performing volume measurements, i.e., the absolute tumor volume and tumor volume regression (Δvolume) at the time of the final BCT treatment predicted residual tumor after treatment and overall treatment failure during follow-up. Volume analysis at first BCT gave promising results however volumetry during the final BCT was superior for predicting residual disease. Indicative for the impact of BCT treatment for treatment outcome in these tumors. As the results of absolute volume analysis were comparable to those of volume regression analysis, absolute volume measurements for the prediction of residual tumor after BCT could be a better option in general practice because it is less time-consuming.

The presence of persistent disease after radiation treatment is a significant predictor of patient survival, early detection of tumors in this group of patients is of clinical importance. For patients with residual disease situated in cervical or adjacent tissue, surgical resection after radiation treatment can be beneficial [4, 5]. To date, most patients receive expectant management until disease recurrence, which might lead to more extensive surgery with increased morbidity or to a situation where the patient might not even be operable because of large tumor size, local involvement of bladder or bowel, distant metastasis, or extensive radiation induced fibrosis. As radiation-induced fibrosis in the target area is lower after completion of EBRT, surgery is an easier option at that time.

Outcome in locally advanced cervix carcinoma has significantly improved through dose escalation enabled by IGABT [14]. The introduction of IGABT resulted in an increase in overall local control (91–95 %) and a reduction of late toxicity by 50–60 % [14]. Our results show that volumetric analysis during radiation treatment can predict treatment failure for individual patients. In these patients therapy could be further tailored to for example surgery and/or adjuvant systemic treatment.

Several groups have performed semi-objective volumetric tumor measurements for the assessment of treatment responses [10–13, 20, 21]. Most of these studies were performed retrospectively with small study populations, the imaging protocols employed differed from study to study, and volume evaluations were obtained using ellipsoid based volume measurements, which are inferior to those obtained by manual and freehand delineation of regions of interest [18].

Mayr et al. reported the results of MRI-based volume analysis during treatment at a single institution [10–13]. Wang and Mayr et al. prospectively assessed reductions in tumor volumes measured by sequential MRI during treatment and found a correlation between the volume reduction ratio and 5-year local control rates [13]. Volume regression after EBRT (40–50 Gy) and not initial tumor volume was the most accurate predictor for local control and disease free survival. They found that a tumor with an initial volume >40 cm^3^ and a residual volume >20 % after EBRT on an MRI scan is a predictor of local treatment failure (Sensitivity 89 % and Specificity 89 %) [13].

In contrast to our study, Mayr et al. reported performing volumetric analysis without BCT applicators in place, necessitating an additional MRI scan. This consequently increases costs and requires the patient to come in the MR unit at a separate time.

The quality of the MRI performed during brachytherapy with the applicator in situ has shown to be sufficient for brachytherapy treatment planning. System based distortion due to the use of a magnetic field are generally under 1-2 mm and are at their lowest in the center of the image. Additional geometric distortions caused by adding a MR-compatible applicator have shown to be nearly undetectable and are at their lowest in the center of the image [22–24]. In general MRI and applicator induced reconstruction uncertainties are smaller than the uncertainties induced by tumor contouring and bowell movement.

As the use of MRI-guided BCT for a cervical carcinoma larger than 5 cm is expected to increase significantly in future as more institutions perform 3D treatment planning instead of 2D X-ray, volumetric analyses based on routine MRI could prove to be more cost effective and convenient [14, 16, 25, 26].

The prevalence of recurrent/persistent local tumors in our dataset (11.4 %) is rather low compared to those previously reported. The low recurrence rate could be due to the lower initial tumor volumes, which in 80 % of our population were <40 cm^3^, or to several other reasons. First, because of the exclusion criteria, patients who did not finish imaging or the treatment protocol because of early progressive disease were excluded. Second, all patients received together with radiation therapy, concurrent chemotherapy or hyperthermia treatment, which resulted in a higher cure rate than that of radiotherapy alone [27, 28]. Third, all patients received MRI-based high-dose-rate intracavitary and interstitial BCT according to the GEC-ESTROO guidelines, which is proven to be superior to 2D-based radiotherapy, especially for cervical carcinomas sized >5 cm [26]. Moreover, we included tumors that were relatively smaller than those reported in other studies.

Our study had some limitations. First, the absence of residual disease was not proven histopathologically in all patients. This was mainly because some patients did not undergo surgery. However, we believe that long-term follow-up in the absence of recurrence is at least as good as performing surgico-pathologic analysis. Especially because in those cases the time interval between pathologic analysis with random biopsies or adjuvant surgery is shorter and it could be that because of this reason microscopic tumors are still existent. Possible this would not have been the case with longer waiting time, because of delayed radiotherapy effects. A second limitation of this study was the small number of patients. MRI-based BCT became the standard protocol in our center in October 2008, which was the main reason for the exclusion of 13 patients who underwent only one MRI scan during BCT.

## Conclusion

Volume measurement at the time of BCT is a potential promising predictor of local tumor response in individual patients, especially when performed during the final BCT. As absolute volumetric analysis yielded results comparable to those of volume regression analysis, a single tumor volume measurement at the time of the last BCT, could already be sufficient to reduce measurement time. We also observed an association between overall treatment failure and local volume response, the potential of assessing local response for identifying individual patients at high risk for overall treatment failure is promising. External validation however in a larger and independent study population is warranted.
